# Global Impacts of Western Diet and Its Effects on Metabolism and Health: A Narrative Review

**DOI:** 10.3390/nu15122749

**Published:** 2023-06-14

**Authors:** Vicente Javier Clemente-Suárez, Ana Isabel Beltrán-Velasco, Laura Redondo-Flórez, Alexandra Martín-Rodríguez, José Francisco Tornero-Aguilera

**Affiliations:** 1 Faculty of Sports Sciences, Universidad Europea de Madrid, Tajo Street, s/n, 28670 Madrid, Spain; vctxente@yahoo.es (V.J.C.-S.); josefrancisco.tornero@universidadeuropea.es (J.F.T.-A.); 2 Psychology Department, Faculty of Life and Natural Sciences, Nebrija University, 28240 Madrid, Spain; abeltranv@nebrija.es; 3 Department of Health Sciences, Faculty of Biomedical and Health Sciences, Universidad Europea de Madrid, C/Tajo s/n, 28670 Villaviciosa de Odón, Spain; lauraredondo_1@hotmail.com

**Keywords:** inflammation, cardiovascular disease, mental disease, metabolic disease, cancer, microbiota, nutrition, physical activity

## Abstract

The Western diet is a modern dietary pattern characterized by high intakes of pre-packaged foods, refined grains, red meat, processed meat, high-sugar drinks, candy, sweets, fried foods, conventionally raised animal products, high-fat dairy products, and high-fructose products. The present review aims to describe the effect of the Western pattern diet on the metabolism, inflammation, and antioxidant status; the impact on gut microbiota and mitochondrial fitness; the effect of on cardiovascular health, mental health, and cancer; and the sanitary cost of the Western diet. To achieve this goal, a consensus critical review was conducted using primary sources, such as scientific articles, and secondary sources, including bibliographic indexes, databases, and web pages. Scopus, Embase, Science Direct, Sports Discuss, ResearchGate, and the Web of Science were used to complete the assignment. MeSH-compliant keywords such “Western diet”, “inflammation”, “metabolic health”, “metabolic fitness”, “heart disease”, “cancer”, “oxidative stress”, “mental health”, and “metabolism” were used. The following exclusion criteria were applied: (i) studies with inappropriate or irrelevant topics, not germane to the review’s primary focus; (ii) Ph.D. dissertations, proceedings of conferences, and unpublished studies. This information will allow for a better comprehension of this nutritional behavior and its effect on an individual’s metabolism and health, as well as the impact on national sanitary systems. Finally, practical applications derived from this information are made.

## 1. Introduction to the Western Diet

Like all species, contemporary humans are genetically adapted to the environment of their ancestors, which conditioned their genetic profile. In terms of nutrition, the introduction of agriculture and animal husbandry about 10,000 years ago has lead to profound changes in the diet and lifestyle of these “modern humans” [1].

**Figure 1:**
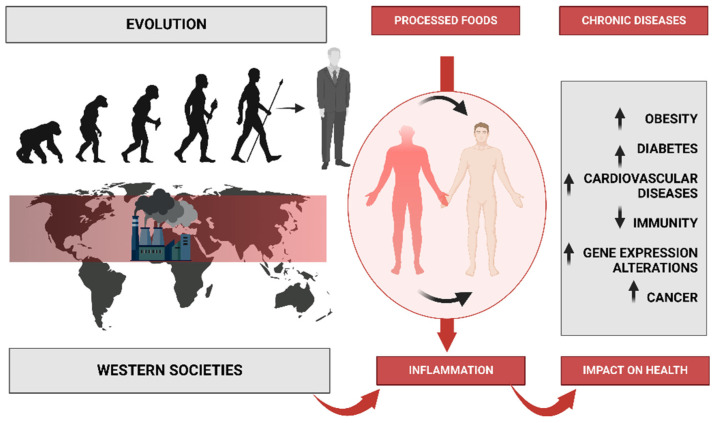
Description of human evolution and the changes in eating patterns caused by increased industrialization and the marketing of processed foods. This leads to poor habits that cause inflammation, which leads to a variety of diseases.

The problem with this is that these changes have occurred too recently on an evolutionary time scale for the human genome to adapt, thus leading to a discordance between our ancient biology and contemporary lifestyle patterns (Figure 1) [2]. Before agriculture and animal husbandry, hominin diets were limited to minimally processed wild plant and animal foods. However, with the domestication of plants and animals, the nutrient characteristics of these foods changed, which accelerated with advancing technology after the Industrial Revolution [3]. With the introduction of agriculture, novel foods were introduced as staples for which the hominin genome had little evolutionary experience. Furthermore, food processing procedures were developed that allowed for combinations of nutrients and foods not previously encountered in hominin evolution [1]. It is crucial to consider not only the nutrient qualities and the types of foods that would have been consumed by preagricultural hominins but also the types of foods and nutrient qualities that could not have been regularly consumed before the development of agriculture, industrialization, and advanced technology. Additionally, it is worth noting that dairy products, cereals, refined sugars, refined vegetable oils, and alcohol make up 72.1% of the total daily energy consumed by all people in the United States [4], but these types of foods would have contributed little or none of the energy in the typical preagricultural hominin diet [5]. Additionally, processed foods also dominate in a typical Western diet, such as cookies, cake, bakery foods, breakfast cereals, and snack foods [6].

### 1.1. Health Consequences, Costs, and Impact

Therefore, there is a dissonance, a change in the food and nutritional model, which reveals a series of epidemiological problems, with an increasing number of many diseases of civilization, including obesity, diabetes, and heart disease [7,8]. In the United States alone, most adults aged 20 and above, around 65%, are either overweight or obese. This has resulted in an estimated 280,184 deaths every year that can be attributed to obesity alone. Cardiovascular diseases (CVD) are prevalent in over 64 million Americans [7], and they remain the leading cause of death, accounting for 38.5% of all deaths in the country [4]. Moreover, 50 million Americans suffer from hypertension and a poor nutritional status, which is a cause of prolonged hospitalization and has a substantial impact on public health cost [9]. Additionally, 11 million have type 2 diabetes and 37 million have high-risk total cholesterol concentrations of 240 mg/dL [9]. In postmenopausal women aged 50 years, around 7.2% have osteoporosis and 39.6% have osteopenia [10]. Additionally, osteoporotic hip fractures have been associated with a 20% excess mortality in the year following the fracture. Cancer is the second leading cause of death in the country, accounting for 25% of all deaths [11]. An estimated one-third of all cancer deaths are due to poor dietary habits and obesity [12]. These figures demonstrate the severe impact of a disruptive diet that moves away from the nutritional model of our ancestors [1], approaching what is known today as the “Western diet”.

Thus, measures must be taken to address these issues, such as promoting healthy eating habits and increasing physical activity. Public health campaigns and initiatives can play a crucial role in educating people about the importance of a balanced diet and an active lifestyle [13]. Policymakers can also implement measures such as food labeling and taxation on unhealthy foods to encourage healthier choices. It is essential to take proactive steps to address these issues and improve the overall health of the nation.

### 1.2. Scientific Evidence

Indeed, recent clinical trials and interventions have shown that adopting dietary treatments with nutritional characteristics similar to those of pre-agricultural and pre-industrial diets can have positive effects on health [14]. These findings are consistent with the evolutionary discordance theory, which suggests that the human body is adapted to certain dietary patterns and that modern diets, which differ significantly from those of our ancestors, may contribute to chronic diseases. Thus, by adopting healthier eating habits that are in line with our evolutionary past, we can potentially reduce the risk of chronic diseases and improve overall health.

However, we have been able to mitigate some of the effects of this discordance through advancements in medicine and technology, which has led to some new challenges given the natural disruption of viral and bacterial processes in modern medicine, such as antibiotic-resistant bacteria [15]. In addition, the impact of human activities on the environment is creating new selective pressures that could lead to further evolutionary changes in the future. As such, understanding the interplay between genetics and the environment is crucial for developing effective strategies to promote human health and well-being in the face of these challenges.

In light of the need for a greater understanding of this nutritional behavior, its effect on an individual’s metabolism and health, and its impact on national health systems, this review was conducted to describe the effect of the Western pattern diet on the metabolism, inflammation, and antioxidant status; the impact on gut microbiota and mitochondrial fitness; the effect cardiovascular health, mental health, and cancer; and the sanitary costs.

## 2. Methods

In this investigation, we performed a comprehensive examination of primary and secondary sources that incorporated scientific articles, bibliographic indexes, and databases such as PubMed, Scopus, Embase, Science Direct, Sports Discuss, ResearchGate, and the Web of Science. We applied MeSH-compliant keywords such as Western diet, inflammation, metabolic health, metabolic fitness, cardiovascular disease, cancer, oxidative stress, mental health, and metabolism to explore articles that were published between 1 January 2003 and 1 March 2023. The following exclusion criteria were used: (i) studies with inappropriate or irrelevant topics not pertinent to the main focus of the review, (ii) Ph.D. dissertations, conference proceedings, and unpublished studies. A team of five review authors meticulously evaluated the titles and abstracts of all collected manuscripts to ascertain their suitability. Studies that utilized outdated data, had irrelevant topics that did not align with the research objectives, or were not in English were eliminated. The same team of five review authors who undertook the study selection independently extracted pertinent data from the selected studies. Subsequently, the outcomes were discussed to create the current comprehensive review. It is worth mentioning that this study’s approach guarantees that the included data are current, relevant, and of high quality, thereby making the findings of this review reliable and useful for future research.

## 3. Nutritional Characteristic of Western Diet

The Western diet has been the subject of much discussion in the field of nutrition. It is a modern dietary pattern that is characterized by high intakes of processed and refined foods, red and processed meats, added sugars, and saturated and trans fats and low intakes of fruits, vegetables, whole grains, and nuts (Figure 1). The Western diet has been linked to a range of chronic diseases, including obesity, type 2 diabetes, cardiovascular disease, and certain cancers. In this paper, we will discuss the nutritional characteristics of the Western diet, its impact on health, and the potential solutions to improve dietary patterns in Western societies [12].

### 3.1. Western Diet Concept

The Western diet is characterized by a high intake of energy-dense, nutrient-poor foods such as fast foods, soft drinks, and highly processed foods, which are high in added sugars, salt, and saturated fats. In contrast, the traditional diets of non-Western countries, such as the Mediterranean diet, are characterized by high intakes of fruits, vegetables, whole grains, legumes, and healthy fats such as olive oil and nuts [16]. The consumption of processed and refined foods is a major characteristic of the Western diet. Processed foods are foods that have been altered from their natural state, typically to increase their shelf life or improve their taste. Examples include fast foods, packaged snacks, and sugary drinks. Refined foods are foods that have had their natural fiber and nutrients removed during processing, such as white flour, white rice, and added sugars [17].

The Western diet is also characterized by high consumption of red and processed meats. Red meat consumption has been associated with an increased risk of colorectal cancer, while processed meat consumption has been linked to an increased risk of colorectal cancer, cardiovascular disease, and type 2 diabetes [18]. Another major characteristic of the Western diet is the high intake of added sugars. Added sugars are sugars and syrups that are added to foods during processing, such as high-fructose corn syrup in soft drinks and table sugar in baked goods. High intake of added sugars has been linked to an increased risk of obesity, type 2 diabetes, and cardiovascular disease [19]. The Western diet is also characterized by high intakes of saturated and trans fats. Saturated fats are found in animal products such as meat, butter, and cheese, while trans fats are found in processed foods such as baked goods and fried foods. High intake of saturated and trans fats has been linked to an increased risk of cardiovascular disease [20].

In order to clarify which countries are exposed to this diet concept, we provide official FAO data. FAO-provided food balance sheets are adjusted for consumer waste and used to calculate a Western dietary similarity index (WSI) for each nation. Based on this information, a ratio of calories from animal foods, oils, lipids, and sweeteners to total calories per capita can be calculated [21,22,23]. A plausible value is WSI = 70, which corresponds to the US WSI in 2013, indicating that the global steady-state WSI will correspond to a dietary pattern in which 70% of calories originate from animal foods, oils and fats, and sweeteners. Iceland (72), Switzerland (72), the United States (70), Australia (69), Sweden (67), Hungary (66), France (66), Austria (66), Germany (66), Denmark (66), the Czech Republic (65), the Netherlands (65), Spain (65), Belgium (65), Finland (64), and New Zealand comprise the “Western diet countries” group (64).

### 3.2. Western Diet-Related Diseases

The Western diet includes low intakes of fruits, vegetables, whole grains, and nuts. These foods are important sources of vitamins, minerals, fiber, and antioxidants, which are essential for optimal health. Low intakes of these foods have been linked to an increased risk of chronic diseases such as obesity, type 2 diabetes, and cardiovascular disease [24]. The Western diet has been linked to a range of chronic diseases and inflammation processes (Figure 1), including obesity, type 2 diabetes, cardiovascular disease, and certain cancers. In the United States, the data reveal alarming trends between 1999 and 2018, with male obesity increasing from 27.5% to 43.0% and severe obesity from 3.1% to 6.0%. In women, the prevalence of obesity rose from 33.4% to 41.9%, and the prevalence of severe obesity rose from 6.2% to 11.5% [25]. Obesity is a major risk factor for a range of chronic diseases, including type 2 diabetes, cardiovascular disease, and certain cancers. The consumption of a Western-style diet has been shown to increase the risk of obesity and related diseases [17]. Type 2 diabetes is another major health concern associated with the Western diet. A high intake of processed and refined foods, added sugars, and saturated and trans fats has been linked to an increased risk of type 2 diabetes [19]. In addition, low intakes of fruits, vegetables, whole grains, and nuts have been associated with an increased risk of type 2 diabetes [24].

Cardiovascular disease is the leading cause of death worldwide, and the Western diet is a major risk factor for this disease. High intakes of saturated and trans fats, added sugars, and salt have been linked to an increased risk of cardiovascular disease [26]. In contrast, diets high in fruits, vegetables, whole grains, and healthy fats such as those found in the Mediterranean diet have been shown to reduce the risk of cardiovascular disease [16]. Certain cancers, particularly colorectal cancer, have also been associated with the Western diet. High intakes of red and processed meats, as well as low intakes of fruits, vegetables, and whole grains, have been linked to an increased risk of colorectal cancer [27].

The availability of processed and ultra-processed foods including sugar, industrial seed oils, and poultry has increased over the past two centuries, while butter/lard/shortening, dairy (primarily whole-fat), fresh fruits, fresh vegetables, and red meat (beef/pork) have decreased. Before 1900, ultra-processed foods were uncommon, but they now make up more than half of the American diet [28]. The gut microbiome is the collection of microorganisms that reside in the digestive tract, including bacteria, viruses, fungi, and other microbes. This complex ecosystem plays an important role in the health of the human body, contributing to various functions such as digestion, immune system regulation, and the synthesis of certain vitamins and other essential nutrients [29]. In the scientific literature, it has been previously stated that one of the main factors, together with physical exercise, that modulates the intestinal microbiota is nutrition [30]. However, due to this, diet impacts the gut microbiome and immune system. Concretely, the Western diet can disrupt the balance and diversity of the gut microbiome leading to dysbiosis, which is a condition characterized by an overgrowth of harmful bacteria and a reduction in beneficial bacteria. Dysbiosis can impair intestinal barrier function, increase intestinal permeability, promote bacterial translocation, and trigger systemic inflammation. Dysbiosis can also affect immune system function, modulate immune cell differentiation, alter immune cell activation, influence cytokine production, regulate immunoglobulin secretion, and modulate immune tolerance [31].

### 3.3. Western Diet’s Impact on Genetics

The Western diet also can influence gene expression and epigenetics. Gene expression refers to the process by which genetic information is translated into functional molecules such as proteins or RNA. Epigenetics refers to the study of heritable changes in gene expression that do not involve changes in the DNA sequence but rather modifications in DNA methylation, histone modification, or microRNA regulation. The Western diet can affect gene expression and epigenetics by altering nutrient availability, cofactor supply, hormonal levels, environmental cues, cellular signaling pathways, transcription factors, chromatin structure, DNA methylation, histone modification, microRNA regulation, feedback loops, genetic variants, gene–environment interactions, intergenerational effects, and transgenerational effects (Figure 1) [32].

The Western diet shifts the co-expression of 445 gene pairs in mice, including small RNAs and transcription factors associated with metabolism and adiposity in humans, and dramatically alters behavior. For example, Western-fed individuals were more anxious and less socially integrated [33]. Similarly, the Western diet limits microbial short-chain fatty acid (SCFA) production in humans, which prevents many of the microbiota-dependent events from occurring, such as histone deacetylation and DNA methylation, and leads to alterations in hepatic gene expression [34]. The Western diet evokes structural and behavioral changes in the resident microbiome, which alters the representation of metabolic pathways in the microbiome and alters microbiome gene expression [35], as well as inducing epigenetic changes in monocytes that affect their inflammatory response and cytokine production [33], altering the expression of genes involved in lipid metabolism and oxidative stress in liver tissue [36], and modulating the expression of genes related to the circadian rhythm and melatonin synthesis in the pineal gland [37]. Similarly, it can also increase gene expression alterations related to a fatty liver (non-alcoholic fatty liver disease).

## 4. Western Diet and Lifestyle

The Western diet has become a global phenomenon and has had significant impacts on human health. Social characteristics such as income, education, and cultural background play a crucial role in shaping dietary habits and preferences. There are some social characteristics of the Western diet, which are related to income and education, since income and education are essential social factors that influence dietary choices. People with higher incomes and more education are more likely to have healthier diets that include more fruits, vegetables, whole grains, and lean proteins. A study by Burrows et al. (2017) found that higher levels of education were associated with a higher intake of fruits and vegetables [38]. Income and education levels are associated with health outcomes related to the Western diet. Lower-income individuals and those with less education are more likely to have poor dietary habits and be at higher risk for chronic diseases such as obesity, type 2 diabetes, and cardiovascular disease (Figure 1). For example, it was found that low-income individuals were more likely to have a higher intake of processed foods and a lower intake of fruits and vegetables [39].

In addition to the Western diet, sedentary lifestyles and low levels of physical activity are also associated with an increased risk of chronic diseases. In a paper that studied 3316 Finnish participants between 25 and 74 years old, it was found that higher levels of physical activity were associated with a reduced risk of type 2 diabetes [40]. Similarly, a study with 88 140 participants aged 40–85 years old found that higher levels of physical activity were associated with a reduced risk of cardiovascular disease [41].

Similarly, social norms can also influence dietary choices. People are often influenced by the behavior of those around them, including family, friends, and peers. One study [42] found that social norms related to healthy eating were positively associated with fruit and vegetable intake among adolescents. Social norms related to healthy eating are associated with better health outcomes. Individuals who perceive social norms to support healthy eating behaviors are more likely to have healthier diets and lower risks for chronic diseases, and adolescents who perceived social norms to support healthy eating had higher intakes of fruits and vegetables and lower intakes of sugar-sweetened beverages [43].

The food environment, including the availability and accessibility of healthy food options, also plays a role in shaping dietary choices. People living in areas with limited access to healthy food options are more likely to have poor dietary habits. A study by Stowers et al. (2020) [44] found that individuals living in food deserts had lower intakes of fruits and vegetables and higher intakes of sugar-sweetened beverages compared to those living in areas with better food access. The food environment is also associated with health outcomes related to the Western diet. People living in food deserts are more likely to have poorer health outcomes due to limited access to healthy food options. A study by Caspi et al. (2012) [45] found that individuals living in food deserts had higher risks for obesity and type 2 diabetes compared to those living in areas with better food access. Understanding these social characteristics is essential for developing effective interventions aimed at promoting healthier dietary habits and reducing the risk of chronic diseases.

## 5. Western Diet and Antioxidant Status

**Figure 2:**
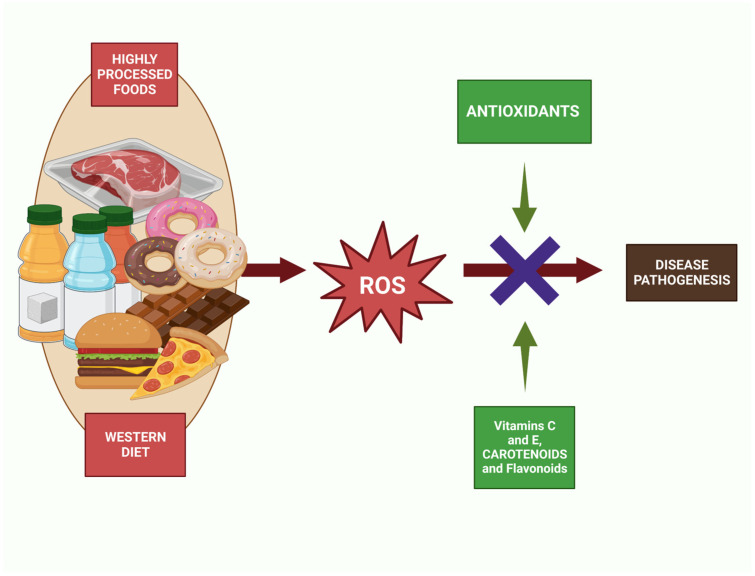
Antioxidant action against the increase in ROS caused by the intake of foods that follow Western diet patterns such as sugary drinks, pastries, or fast food.

The Western dietary pattern has been associated with an increased risk of chronic diseases, and one of the mechanisms by which the Western diet may contribute to the development of these diseases is through oxidative stress [46]. Oxidative stress occurs when there is an imbalance between the production of reactive oxygen species (ROS) and the body’s antioxidant defense system (Figure 2). In this process, a free radical can be defined as any molecular species capable of independent existence that contains an unpaired electron in an atomic orbital. The presence of an unpaired electron gives rise to certain common properties that most radicals share. Many radicals are unstable and highly reactive. They can either donate or accept an electron from other molecules, so they behave as oxidizers or reductants [47]. As a consequence of an imbalance between free radical production and antioxidant defenses, oxidative stress is associated with injury to a broad spectrum of molecular species, including lipids, proteins, and nucleic acids [48]. Due to important macromolecules being attacked by free radicals, cell injury and a disruption of homeostasis develop [49]. An antioxidant is a molecule that is stable enough to donate an electron to a free radical and neutralize it, thereby reducing its destructive potential [50]. These antioxidants delay or prevent cellular injury primarily due to their ability to neutralize free radicals. These low-molecular-weight antioxidants can interact with free radicals and terminate the chain reaction without causing injury to vital molecules. Some of these antioxidants, such as glutathione, ubiquinol, and uric acid, are produced by the body’s normal metabolism [51]. There are additional milder antioxidants in the diet. Although there are numerous enzyme systems in the body that neutralize free radicals, the most important micronutrient (vitamin) antioxidants are vitamin E (tocopherol), vitamin C (ascorbic acid), and beta-carotene. Because the body cannot produce these micronutrients, they must be consumed [52]. In summary, antioxidants can neutralize ROS and prevent oxidative damage. However, the Western diet is generally low in these antioxidant-rich foods and high in pro-oxidant substances such as omega-6 fatty acids and advanced glycation end products (AGEs) [53].

Since the diet type analyzed in this paper is deficient in antioxidants, the problem is exacerbated [54,55].The main sources of dietary antioxidants are fruits, vegetables, whole grains, and nuts. However, the Western diet is deficient in these foods, which means that individuals following this dietary pattern are more likely to have low levels of antioxidants [56]. Studies have shown that individuals following the Western diet have lower levels of the previously mentioned vitamin C, vitamin E, and beta-carotene than those consuming a healthy diet [57,58]. For example, a study conducted on a sample of adults in the United States found that those following the Western diet had lower serum levels of vitamin C and vitamin E than those following a healthy diet [59]. Similarly, a study conducted on Australian adults found that those consuming a Western diet had lower serum levels of beta-carotene than those consuming a healthy diet [60].

Several studies have highlighted the importance of a diet rich in antioxidants for maintaining optimal health (Figure 2) [61]. A high intake of fruits and vegetables, which are rich sources of antioxidants, has been associated with a reduced risk of chronic diseases, [62] including cardiovascular disease, cancer, and neurodegenerative disorders [63,64]. In contrast, a Western diet that is low in fruits, vegetables, and whole grains and high in saturated fats, sugar, and processed foods has been linked to a higher risk of chronic diseases. A study conducted in the United States found that individuals consuming a Western diet had a higher risk of heart disease, diabetes, and stroke than those consuming a healthy diet [65]. Furthermore, the Western diet not only lacks essential antioxidants but also contains pro-oxidant compounds that increase the production of free radicals and oxidative stress in the body [66]. A study conducted on a sample of healthy adults found that consuming a high-fat meal induced oxidative stress, increased inflammation, and decreased antioxidant capacity in the body [67]. The study also found that consuming a meal rich in fruits and vegetables before the high-fat meal reduced oxidative stress and inflammation and improved antioxidant capacity.

Studies have found that the Western diet is associated with lower antioxidant status in the body, as measured by levels of antioxidant enzymes and biomarkers [68]. This may contribute to the development of chronic diseases, as low antioxidant status has been linked to increased oxidative stress and inflammation [69]. Therefore, improving antioxidant status through dietary changes may have potential health benefits, particularly in individuals with a high intake of the Western diet. One study found that increasing the consumption of fruits and vegetables, which are high in antioxidants, was associated with a lower risk of mortality from cardiovascular disease and cancer [70]. Another study found that supplementation with antioxidant vitamins reduced the risk of cardiovascular events in high-risk individuals [71].

Moreover, the Western diet may exacerbate oxidative stress in the body by reducing the activity of endogenous antioxidants such as glutathione and superoxide dismutase (Figure 2) [72]. Oxidative stress occurs when the body’s antioxidant defenses are overwhelmed by the production of reactive oxygen species (ROS) and reactive nitrogen species (RNS). These free radicals can damage DNA, proteins, and lipids, leading to cell dysfunction and death [73,74,75]. Oxidative stress has been implicated in the pathogenesis of numerous diseases, including cancer, neurodegenerative disorders, and cardiovascular disease [76]. The Western diet is a major contributor to oxidative stress [73,74,75]. High intake of processed foods and sugar-sweetened beverages has been linked to increased production of ROS and RNS (Figure 2) [10]. These foods are often high in refined carbohydrates and saturated and trans fats, which can promote inflammation and increase the production of ROS and RNS [77]. Red and processed meat, another hallmark of the Western diet, are also associated with oxidative stress. The heme iron in red meat can catalyze the formation of ROS, leading to oxidative damage [78]. Additionally, processed meats contain nitrates and nitrites, which can react with other compounds in the body to form RNS [79]. Refined grains, another staple of the Western diet, are low in fiber and other important nutrients, which can increase inflammation and oxidative stress [80]. High intake of refined grains has been associated with increased levels of markers of oxidative stress in the blood [81].

In a study conducted on overweight and obese adults, the consumption of a Western diet for eight weeks was found to reduce the activity of superoxide dismutase and increase oxidative stress markers in the body [82]. These findings suggest that a Western diet may increase the risk of chronic diseases by promoting oxidative stress and reducing the body’s ability to defend against it. Low levels of antioxidants can have significant implications for health. Oxidative stress, which results from an imbalance between free radicals and antioxidants in the body, can damage cells, proteins, and DNA. This damage can lead to chronic diseases such as cancer, cardiovascular disease, and neurodegenerative diseases [83]. In addition, low levels of antioxidants have been associated with an increased risk of inflammation and oxidative damage to the body’s tissues [76].

In addition to consuming a diet rich in antioxidants, other lifestyle factors can impact antioxidant status. Physical activity has been shown to increase antioxidant capacity in the body, reduce oxidative stress, and improve overall health [84]. It was found that individuals who engaged in regular physical activity had higher levels of antioxidant enzymes, such as superoxide dismutase and glutathione peroxidase, compared to sedentary individuals [85]. Additionally, smoking and alcohol consumption have been associated with decreased antioxidant levels in the body and increased risk of chronic diseases [86,87].

In conclusion, the Western diet is associated with a lower antioxidant status compared to healthy dietary patterns, and this may increase the risk of chronic diseases. A diet rich in fruits, vegetables, whole grains, nuts, and legumes can provide essential nutrients, including antioxidants, that are critical for optimal health. Therefore, it is recommended that individuals adopt a healthy dietary pattern that emphasizes plant-based foods and limits the intake of processed and high-fat foods to promote good health and reduce the risk of chronic diseases.

## 6. Western Diet and Inflammation

High consumption of saturated fats, processed foods, and refined sugars is characteristic of the Western diet and has been associated with persistent low-grade inflammation. Critical in the body’s defense against infections and injuries, inflammation is a complicated process involving the immune system [88]. Specifically, chronic inflammation can lead to the development of several chronic diseases, such as cardiovascular disease, type 2 diabetes, and cancer (Figure 1) [89,90]. In this regard, research has demonstrated that Western diet patterns, which are also associated with a high intake of red and processed meats, refined cereals, and caffeinated beverages, promote elevated levels of *C*-reactive protein (CRP), a well-established marker of inflammation according to previous research [91]. Additionally, authors have also reported that the Western diet was associated with increased levels of CRP and other inflammatory markers, such as interleukin-6 (IL-6) and tumor necrosis factor-alpha (TNF-α) [92]. In this case, the WD results in a persistent metabolic inflammatory state [66].

Multiple mechanisms, including oxidative stress, intestinal microbiota dysbiosis, and immune dysregulation, have been also shown to contribute to the pro-inflammatory effects of a Western diet (Figure 1). When there is an imbalance between the production of reactive oxygen species (ROS) and the body’s ability to detoxify, oxidative stress occurs [74,93]. Thus, saturated and trans fats, which can increase ROS production and impair antioxidant defenses, are the main promoters of oxidative stress creation [73]. This can result in damage to cells, tissues, and organs, and the release of pro-inflammatory cytokines, such as IL-6, and TNF-α [94]. ROS are highly reactive molecules that are formed as a byproduct of cellular metabolism, and their production is tightly regulated in the body. However, oxidative stress conditions injure cells and tissues [95]. This damage can contribute to the development of chronic diseases such as cardiovascular diseases, diabetes, and cancer [95].

In addition to providing evidence along these lines, rodent studies may be pertinent to dietary changes in humans. Concretely, recent studies found that the consumption of a high-fat diet led to increased production of ROS in rats [96]. It was also shown that antioxidants lose their efficacy in an inflammatory state, making this a progressive, hard-to-control rise in oxidative stress [97]. These findings suggest that the consumption of a Western diet may contribute to the development of chronic diseases through increased ROS production. Similarly, processed and fried foods, baked goods, and high-fat dairy products have been shown to increase LDL cholesterol levels and contribute to the development of cardiovascular diseases [98]. Thus, high-fat diets are particularly harmful, as they not only increase LDL cholesterol levels but also decrease HDL cholesterol levels, leading to an unfavorable lipid profile [98].

Given the preceding, all of the evidence indicates that high-fat diets result in immune dysregulation. Specifically, low-grade inflammation is promoted by an imbalance in the actions of the immune system. In the Western diet, sugar and fatty acids can either be pro-inflammatory—such as omega-6—and present in high numbers, or they can be anti-inflammatory—such as omega-3 or antioxidants—and present in lesser proportions [99]. This can promote the activation of immune cells, such as macrophages and T cells, and the release of pro-inflammatory cytokines, such as IL-1β, IL-6, and TNF-α [100]. The consumption of high levels of saturated and trans fats found in the Western diet can activate Toll-like receptors (TLRs) on immune cells, leading to the production of pro-inflammatory cytokines and chemokines [101]. TLRs are important mediators of inflammatory pathways that detect pathogen-associated molecular patterns (PAMPs) and damage-associated molecular patterns (DAMPs) and initiate an immune response [102]. In response to a Western diet, the TLR pathway is activated by the high levels of endotoxins and other pro-inflammatory molecules present in the diet. This activation leads to the production of pro-inflammatory cytokines, such as TNF-α, IL-6, and interleukin-1 beta (IL-1β), which are involved in the pathogenesis of chronic diseases [101].

To continue, it is relevant to note that the method of cooking is also thought to alter or may affect metabolic processes. In relation to this, advanced glycation end products (AGEs)—compounds created when food is cooked at very high temperatures—are abundant in the Western diet. When AGEs bind to the RAGE receptor, pro-inflammatory cytokines are released [103]. AGEs are a group of complex molecules formed by the non-enzymatic reaction between reducing sugars and amino acids or proteins. This reaction is known as the Maillard reaction and occurs naturally in the body as part of normal metabolism, but also through external sources such as cooking methods, particularly high-temperature cooking, and the processing of food [104]. AGEs are known to accumulate in tissues over time and contribute to the development of various diseases [105,106]. AGEs are formed through the non-enzymatic reaction between reducing sugars and amino acids and are present in high levels in processed foods, particularly those that are high in fat and sugar [103]. The Western diet is high in AGEs due to the consumption of processed and refined foods, which are often cooked at high temperatures, leading to the formation of AGEs [107]. These AGEs can be found in various foods, including meat, dairy, and baked goods, which are staple items in the Western diet [103]. It was found that participants who consumed a high-AGE diet for four weeks had increased markers of inflammation and oxidative stress, which are known to contribute to the development of chronic diseases such as diabetes and cardiovascular diseases [108]. AGEs can activate the receptor for advanced glycation end products (RAGE), which is expressed on the surface of many cell types, including endothelial cells, macrophages, and adipocytes. RAGE activation leads to the production of pro-inflammatory cytokines and oxidative stress, which are involved in the pathogenesis of chronic diseases [103].

Regarding gut diseases, dysbiosis, or an imbalance of the intestinal microbiota, has been linked to the Western diet and may contribute to inflammation. Dysbiosis of the gut microbiota describes a state of discord between the microbes normally found in the digestive tract and their host. The Western diet lacks the fiber and prebiotics necessary to foster the development and variety of good gut flora [109]. Instead, the diet is high in fat, sugar, and salt, which can promote the growth of harmful bacteria and fungi. This can result in gut inflammation, increased gut permeability, and the release of bacterial endotoxins, such as lipopolysaccharides (LPS), into the bloodstream. These endotoxins can activate the immune system and promote the release of pro-inflammatory cytokines, such as IL-1β and IL-18 [110]. Previous authors found that the Western diet was associated with decreased gut microbial diversity and increased levels of pro-inflammatory bacteria, such as Proteobacteria [111]. The dysbiosis induced by the Western diet can lead to increased intestinal permeability, allowing bacterial endotoxins to enter the bloodstream and trigger inflammation [112].

## 7. The Effect of Nutrition and the Western Diet on the Intestinal Microbiota

Based on the above, our knowledge reveals that eating a lot of processed, high-calorie meals is not only associated with an increased risk of developing conditions including diabetes, obesity, heart disease, but also gut dysbiosis. Concretely, the high meat consumption related to the Western diet increases Bacteroides, Alistipes, and Bilophila (associated with pathological processes such as atherogenesis) and decreases Bifidobacterium, Roseburia, Eubacterium, and Ruminococcus [113]. Additionally, the Firmicutes/Bacteroidetes ratio has been frequently considered a plausible marker for obesity over the past decade and a parallel of Western diet patterns [114]. In this regard, the abundance of Firmicutes in the gastrointestinal microbiota of healthy individuals ranges from 11% to 95%, while that of Bacteroidetes ranges from 0.6% to 86.5%. However, Kasai et al. reported more accurately that Firmicutes comprised 37.0 ± 9.1% of non-obese and 40.8 ± 15.0% of obese individuals, whereas Bacteroidetes comprised 44.0 ± 9.8% of non-obese and 37.0 ± 14.0% of obese individuals [115]. These alterations in bacterial composition and/or diversity are typically associated with alterations in the microbiota’s metabolic profile, which also impact host health [116]. However, the relative abundance of the phyla Firmicutes and Bacteroidetes is highly variable among members of the same population [117]. This is likely due to the influence of numerous harmful lifestyle factors, such as the Western diet [118], low physical activity, dietary additives and contaminants, or antibiotic consumption. This may explain the contradictory results observed when comparing the microbiota of normal-weight and obese subjects, making it challenging to correlate the ratio of Firmicutes to Bacteroidetes with a particular health status [117]. Although the intestinal microbiota may contribute to the development of obesity, there is insufficient evidence to support a link between obesity and changes in the Firmicutes/Bacteroidetes ratio [119].

In addition, the combination of a high intake of fat and sugar with a low intake of fiber, fruits, and vegetables is associated with chronic inflammation. Consequently, inflammation is an important underlying mechanism connecting the Western diet to chronic diseases [120]. Regarding this, Bolte et al. demonstrated through long-term diet protocol research that higher abundances of Firmicutes, Ruminococcus species of the Blautia genus, and endotoxin synthesis pathways were consistently associated with processed and animal-derived diets. In contrast, plant and fish diets were found to be positively associated with short-chain fatty acid-producing commensals and nutrient metabolism pathways [121].

Similarly, authors have suggested that the Western diet promotes inflammation in the body by increasing the levels of pro-inflammatory molecules such as cytokines and *C*-reactive protein [122]. Furthermore, the intake of saturated and trans fats, as well as refined carbohydrates, typical in this diet may trigger the release of inflammatory mediators [123]. In contrast, a Mediterranean diet, rich in fruits and vegetables, whole grains, and lean protein sources such as fish, can help reduce inflammation and protect against chronic diseases [124]. Illescas et al. found in a meta-analysis that diets and gastrointestinal maladies are linked to inflammation and cancer, highlighting the distinctive characteristics of the bacterial population associated with the Mediterranean diet. In particular, the microbiota of subjects following a MD was enriched with pro-anti-inflammatory bacteria. For the first time, studies revealed an increase in Akkermansia and a decrease in Fusobacterium with a MD, even below the levels observed in healthy individuals with no defined diet without a diet plan. Fusobacterium is a known pathogenic bacterium associated with malignancy, whereas Akkermansia is indicative of a healthy gastrointestinal tract [125]. One of the main reasons for this is the anti-inflammatory capacity provided by the antioxidants in this diet. These foods are high in antioxidants, which can neutralize free radicals and prevent oxidative damage to cells. Additionally, they contain anti-inflammatory compounds, such as polyphenols and omega-3 fatty acids, which can help reduce inflammation in the body [126].

Dysbiosis and the process of chronic inflammation, as well as the profile and nutritional quality of these typical Western foods, occur simultaneously. Intestine dysbiosis is a condition that occurs when the natural equilibrium of microorganisms in the intestine is disturbed, resulting in an overgrowth of harmful bacteria or a reduction in beneficial bacteria [127]. The foods we consume provide not only the essential nutrients required for our health but also create an environment for the growth and sustenance of gut bacteria. The composition of our diet plays a significant role in determining the type of bacteria that flourish in our gut. As the importance of gut microbiota in maintaining good health has been recognized, there has been growing interest in studying the impact of diet on gut microbiota [111,128,129,130]. Specifically, the Western diet model is hypothesized to increase proinflammatory cytokines, disrupt the epithelial barrier, and alter the intestinal microbiota (with an increase in Bacteroides and Enterobacteriaceae and a decrease in Bifidobacterium and Lactobacillus), thereby promoting low-grade chronic inflammation in the gut [131].

One of the groups of foods that seem to have a greater impact on the intestinal microbiota and that receive the most attention in the scientific literature are ultra-processed foods [132]. These comprise food products made from substances extracted from whole foods that are then combined with various additives, such as preservatives, flavorings, and sweeteners [133]. Ultra-processed foods, such as soft drinks and savory snacks, are highly appealing due to their long shelf-life, affordability, and convenience for consumption anytime and anywhere [134]. Their consumption is alarmingly high in many high-income countries. For instance, in France [135], ultra-processed food contributes to 29.1% of total energy intake, while in Australia and the United States, it accounts for 42% and 57.9% [136], respectively. The negative effects on the gut microbiota [6] have been studied by authors such as Zinöcker MK and Lindseth IA. Authors suggest that ultra-processed foods that contain high levels of added sugars, unhealthy fats, and sodium alter the gut microbiota, leading to a reduction in beneficial bacteria and an increase in harmful bacteria. Moreover, these foods are low in fiber, which is a critical nutrient that promotes the growth of beneficial gut bacteria. In this regard, a recent meta-analysis concluded that vegetable consumption was associated with a lower risk of ulcerative colitis alone, whereas fruit consumption was associated with a lower risk of both Crohn’s disease (CD) and ulcerative colitis [137]. However, authors also suggest that the emulsifiers and additives used in the production of ultra-processed food may have harmful effects on gut microbiota, including inflammation and increased permeability of the gut lining. Additionally, some studies suggest that the consumption of ultra-processed foods may lead to changes in brain activity and behavior, including addiction-like behaviors, which can contribute to overconsumption and further health problems [138]. Analysis of the feces of adults consuming aspartame and acesulfame K in a recent cross-sectional clinical study with 31 participants did not reveal an increase in bacterial abundance, but rather a decrease in bacterial diversity compared to non-consumers. As mentioned, the data may be inconsistent; the median Bacteroidetes:Firmicutes ratio did not change in seven participants who consumed 1.7–33.2 mg/day of acesulfame K for four days, but bacterial diversity statistically differed from that of non-consumers [139,140].

In contrast, vegetarian or plant-based diets may be beneficial to the gut microbiome [141]. Dietary polyphenols are plant-based natural chemicals found in vegetables, grains, fruits, coffee, and tea [142]. These organic molecules are a complex and heterogeneous class of substances characterized by hydroxylated phenyl groups, which are present in very small concentrations in the Western diet [143]. They are frequently divided into flavonoids and non-flavonoids according to their chemical structure and complexity. The influence of dietary polyphenols on gut ecology and the mechanisms behind the effects on intestinal and extraintestinal illnesses has recently been explored. Various research works have discovered antioxidant, antidiabetic, anticarcinogenic, neuroprotective, anti-inflammatory, cardioprotective, antibacterial, antiadipogenic, and other properties in various dietary polyphenols [144].Concretely, epidemiological studies have found links between the consumption of polyphenol-rich foods such as vegetables and drinks and illness prevention. These results have been seen in certain types of cancer, cardiovascular disease, type 2 diabetes, osteoporosis, pancreatitis, gastrointestinal problems, lung damage and neurodegenerative diseases [145].

Although the gut microbiota can be widely utilized as a tool for food safety assessment, more research is needed to better understand the relationship between the gut microbiota and food consumption. While there is mounting evidence for the potential link between gut microbiota and health outcomes, further studies are necessary to establish a causal relationship.

## 8. Western Diet and Mitochondrial Fitness

**Figure 3:**
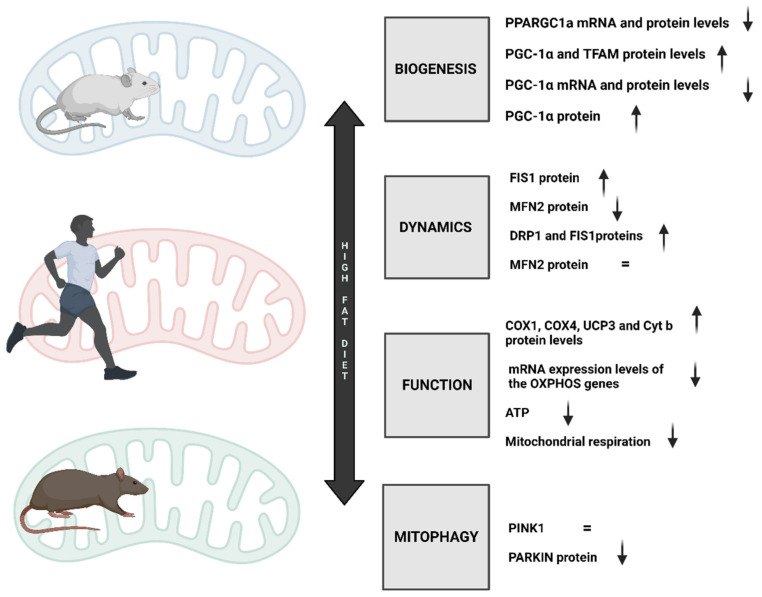
Changes in the most essential mitochondrial processes, such as mitochondrial functioning, dynamics, biogenesis, and mitophagy, as a result of consuming a diet rich in typical Western foods.

Individuals’ diets consist of mixtures of foods and beverages (referred to as foods for simplicity). However, the precise combination of foods that constitutes a healthy diet is context-dependent and influenced by several cultural, economic, and other variables [146]. In recent decades, global food habits have shifted towards increasingly Westernized and less healthy diets. Based on the World Health Organization’s (WHO) worldwide nutrition report, while a portion of the world’s population suffers from starvation, the other portion suffers from obesity and its accompanying problems [147]. With the right proportion, content, quantity, and presence of macronutrients, micronutrients, and bioactive chemicals, a balanced diet counteracts these severe situations. Unfortunately, little is understood about how these components affect human health. These nutrients destined to nourish our bodies, tissues, and cells must first reach the mitochondria, where they are broken down into CO_2_ and H_2_O to produce energy. Mitochondria are the cell’s powerhouse and are primarily responsible for nutrition metabolism [148]. Nevertheless, they are also the primary cause of oxidative stress and apoptosis-mediated cell death [149]. However, mitochondrial performance might be “improved” by physical activity, which improves their integrity, efficiency, and dynamic adaptability to stressors, thus, “mitochondrial fitness” [150]. Mitochondria are extremely dynamic organelles, and their bioenergetics are directly linked to the fusion/fission equilibrium. Fusion activities relate to the enhancement of mitochondrial function, whereas fission processes are associated with the elimination of dysfunctional mitochondria. These processes are influenced by the consumption of a variety of foods, which gives cells several stimuli. Concretely, they are influenced by the energy substrate availability caused by food quality and diet timing. In this regard, it is well acknowledged that different dietary fat sources have distinct effects on the dynamics and bioenergetics of the mitochondria, including mitophagy (Figure 3) [6]. For instance, obesity-related disorders such as insulin resistance and non-alcoholic fatty liver disease are greatly influenced by mitochondrial dysfunction [151].

The traditional Western diet (WD) pattern’s dietary traits are specifically a high intake of saturated fats and omega-6 fatty acids, a decreased intake of omega-3 fats, an abundance of processed carbohydrates, and an overuse of salt [8]. Drinks account for 47% of the added sugars in this surplus, which amounts to more than 13% of the daily calorie intake [7]. Thus, considering the WD as a high-fat diet (HFD), one of the most important pathophysiological symptoms of HFD is insulin resistance, which causes obesity, among other diseases [7]. One of the well-explained pathophysiological changes that an HFD promotes is impaired mitochondrial biogenesis (Figure 3). Concretely, excessive calorie consumption causes tissue hypertrophy and adipocyte hyperplasia in white adipose tissue [152]. The subsequent manifestations increase lipolysis in fat cells and ultimately result in raised amounts of free fatty acids (FA) in circulation. Enhanced lipid catabolism and energy production via the Krebs cycle have already been established as outcomes of increased FA oxidation in the mitochondria [152]. The adipocyte-secreted hormones adiponectin, leptin, acylation-stimulating protein, and resistin play a crucial role in regulating mitochondrial biogenesis and insulin sensitivity [153,154]. In this regard, adiponectin physically binds to its receptors during HFD intake, activating AMP-activated protein kinase (AMPK), which ultimately stimulates glucose uptake and FA oxidation. Moreover, AMPK has been linked to the control of PGC-1α, the principal regulator of mitochondrial biogenesis [155]. According to studies on animals, PGC1 α mRNA and protein levels in skeletal muscle were likewise decreased in C57Bl/6 mice fed an HFD for three weeks [156]. Diastolic dysfunction and poor mitochondrial function were associated in a different investigation. Nevertheless, PGC-1 and mitochondrial transcription factor A (TFAM) protein levels were elevated in the gastrocnemius muscle of male and female Wistar rats that were 2 months old and fed an HFD for 26 weeks. The same study hypothesized that as male rats showed a larger rise in PGC-1 α and TFAM levels than female rats, the effects of an HFD on mitochondrial biogenesis may be sex-dependent [154]. As previously mentioned, a prolonged HFD affects the availability of substrates that mitochondria can use; as a result, mitochondria need to adapt their molecular machinery to generate ATP effectively and sufficiently, especially in high-energy consuming tissues, such as the brain and myocardium. In HFD-fed (60% kcal) rats, Chen et al. reported that reduced complex I-III and citrate synthase activities have been found, which are significant findings that support compromised cardiac function. Similarly, rats showed decreased mitochondrial respiration activities [157], which also supports compromised cardiac function. Likewise, it has been demonstrated that feeding male Wistar rats an HFD for 4 weeks increases their levels of OXPHOS-related proteins such as cytochrome c oxidase (COX)I, COXIV, uncoupling protein (UCP) 3, and cytochrome b [158]. Despite the fact that some studies have shown an increase in the OXPHOS complex, perhaps as a compensatory mechanism to enable mitochondria to produce more energy, an HFD appears to impair mitochondrial function, affecting cellular energetics and ATP turnover [152].

Although an HFD has been shown to lead to insulin resistance, impaired mitochondrial function, and increased production of reactive oxygen species (ROS), much more needs to be known about the dysregulated expression profile of proteins involved in mitochondrial dynamics. Regarding the proteins involved in mitochondrial dynamics, an HFD diet caused mitochondrial fusion protein 2 (MFN2) to drop and proteins involved in fission processes to rise (Drp1 and FIS1) [159]. In this regard, Chen et al. also confirmed that male Sprague Dawley rats fed an HFD (60 percent kcal) showed increased mitochondrial FIS1 levels [16]. Consistent with this, it was also found that healthy male C57BL/6 mice that were 4 weeks old and fed an HFD for 16 weeks showed reduced relative expression of MFN2, but higher levels of mitochondrial DRP1 expression [160]. Lionetti et al., according to electron microscopy and immune activity analysis, suggested that this type of diet also caused a shift towards fission in skeletal muscle. These results are consistent with earlier reports that type 2 diabetic patients and obese Zucker rats have decreased MFN2 expression in their skeletal muscles [159]. Furthermore, in the work of Jheng et al., differentiated C2C12 skeletal muscle cells exposed to saturated fatty acids have been shown to trigger fission processes in vitro that are linked to mitochondrial dysfunction [161]. In addition, high-glucose (HG) treatment has been linked to mitochondrial fragmentation and an increase in fission processes in myoblasts and a rat liver cell line. It has been hypothesized that this fragmentation would increase the total surface area of metabolically active organelles, increasing the accessibility of metabolic substrate to carrier proteins. As a result, it is possible to hypothesize that HFD-induced mitochondrial fragmentation is a cellular adaptation to increase mitochondrial intake and oxidize excess dietary fatty acids, which would lead to increased ROS production [159,162].

Regarding mitophagy during HFD, it has been found that in healthy, non-obese, sedentary males and 10 endurance-trained male runners, the protein content of total PTEN-induced kinase 1 (PINK1) in skeletal muscle was found to be similar between groups before and after a high-fat meal, indicating that mitophagy is not required for metabolic flexibility in the healthy population [163]. However, it has been demonstrated that mice fed an HFD (60 kcal% fat) for two months display mitophagy, which is followed by decreased mitochondrial abundance (mtDNA/nuDNA) [164]. Additionally, a decrease in PARKIN in mitochondria that were supposed to be recruited by PINK1 has been demonstrated [165]. Although mitochondria can readily adapt to exogenous parameters and maintain their proper functionality to generate sufficient energy for cellular demands, these organelles are sensitized to variations in substrate availability due to different dietary habits, followed by molecular rewiring. This orchestrates alterations in mitochondrial function, regulation of biogenesis and mitophagy, and ‘self-recycling’ via fusion and fission in mitochondria. In conclusion, a high-fat diet impairs mitochondrial function and fusion while inducing fission.

## 9. The Effect of Western Diet on Cardiovascular Health

**Figure 4:**
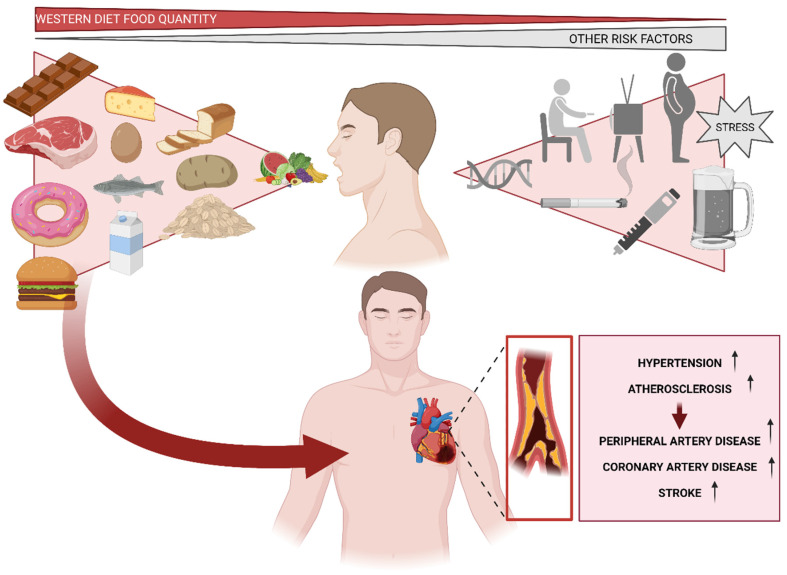
Cardiovascular risks associated with a high-fat diet and other relevant factors that may influence their development, such as genetics, obesity, sedentary lifestyle, diabetes, and alcohol or tobacco consumption.

In Western societies, cardiovascular disease (CVD) is the leading cause of death, with coronary heart disease (CHD) accounting for more than 50% of cases. Numerous risk factors, including family history, diabetes, hypertension, obesity, smoking, and inactivity, contribute significantly to the overall cardiovascular risk (Figure 4). Recent data indicate an interesting gradient in the incidence, morbidity, and mortality of cardiovascular disease across the socioeconomic status spectrum, as defined by educational level, occupation, or income [166]. Personal characteristics, such as physical inactivity, smoking, stress, and alcohol consumption, only account for 13.6% of the SE differences in CVD morbidity and 19.5% of the SE differences in CVD mortality (Figure 4) [167]. Despite the above-mentioned established facts, dietary interventions are much less frequently used in the management of cardiovascular disease than pharmacological and procedural interventions. Nutritional guidelines recommend eating a diet high in fish [168], whole grains [169], vegetables and fruits [170], legumes [171], and nuts to reduce the risk of developing atherosclerotic cardiovascular disease (ASCVD). To lower the risk of ASCVD, dietary monounsaturated and polyunsaturated fats should be substituted for saturated fat, along with sodium, cholesterol, processed meats, refined carbohydrates, and sweetened beverages [172].the consumption of Western-type diets (WDs), which exclude fiber, vitamins, and minerals, has increased over the past few decades in Westernized societies. WDs include processed foods, “fast food”, convenience foods, snacks, and sugary soft drinks (Figure 4). With the spread of these food items and their consumption from high- to low-income countries, there has also been a parallel rise in diseases, as mentioned for CVD, linked to the Western diet [77,173].

A variety of conditions affecting the heart and blood vessels are referred to as CVD, including hypertension, strokes, atherosclerosis, peripheral artery disease, and vein diseases [174]. The combination of unhealthy nutritional habits and habits typical of Western societies creates an environment that is highly prone to cardiovascular system failure (Figure 4) [175,176]. Concretely, ASCVD is an inflammatory disease that contributes significantly to the incidence and mortality of CVD. Libby, on the one side [177], and Mozzaffarian et al. [178], on the other, pointed out in a study based on atherosclerosis therapies that oxidative stress and systemic inflammation are modifiable by nutrition; however, excessive energy intake and physical inactivity contribute to the secretion of pro-inflammatory cytokines [179]. Inflammatory processes involve the subendothelial region of the arterial wall, where lipids and macrophages loaded with lipids accumulate [180]. Concretely, Virmani et al. specified that early stages of atherosclerosis are characterized by the internalization of lipids in the intima, primarily low-density lipoproteins (LDL), which results in endothelial dysfunction. Endothelial dysfunction disruption promotes inflammatory process, emboli, and multiple pathological outcomes, along with calcifications, stenosis, burst, and hemorrhage. Advanced atherosclerosis manifests clinically as coronary heart disease, ischemic stroke, peripheral artery disease, heart failure, or sudden death [181,182]. However, Yubero-Serrano’s findings demonstrate that the Mediterranean diet, especially in CHD patients with severe endothelial dysfunction, controls endothelial function better than a low-fat diet and is linked with a better balance of vascular homeostasis [183].

Current scientific evidence demonstrates that chronic inflammation plays an important role in the pathogenesis of coronary artery disease (CAD), including the initiation and progression of atheroma plaque and rupture, as well as post-angioplasty and restenosis. *C*-reactive protein (CRP), interleukin (IL)-1, IL-6, IL-8, IL-1, IL-18, monocyte chemoattractant protein (MCP)-1, and tumor necrosis factor (TNF α), among others, are the primary mediators of CAD development. Additionally, the results of Usui et al.´s study show that IL-17 is crucial for the emergence of moderate atherosclerosis and shed new light on IL-17’s function in the pathogenesis of atherosclerosis [184]. Moreover, these mediators are considered potential biomarkers of inflammation, and their expression may be correlated with the severity of coronary artery disease [185,186]. Oikonomou et al. provided further evidence assessing 118 stable symptomatic patients and concluded that the Western diet (increased intake of fat, red meat, and carbohydrates and low consumption of fruits and green leafy vegetables) was predictive of severe CAD [187].

Well-established knowledge suggests that Western dietary patterns, in comparison to healthier dietary patterns such as the “Mediterranean diet” (MeDiet), are associated with an increase in the production of proinflammatory cytokines and a decrease in the production of anti-inflammatory cytokines [188,189,190]. Therefore, greater adherence to healthier eating habits, which include consuming more fruits, vegetables, legumes, nuts, and whole grains, may lessen low-grade inflammation and thereby prevent cardiovascular disease [191,192]. A cohort study carried out by Gao et al. identified food-based dietary patterns that operate through excess energy intake and explained high variability in energy density, free sugars, saturated fat, and fiber intakes to investigate their association with total and fatal CVD and all-cause mortality [193]. High intakes of chocolate and sweets [194,195], butter, and low-fiber bread and low intakes of fresh fruits and vegetables comprised the predominant dietary pattern and were positively associated with total CVD [193]. In contrast, current evidence demonstrates that healthy dietary patterns are characterized by a high intake of fiber, antioxidants, vitamins, minerals, polyphenols, and monounsaturated and polyunsaturated fatty acids and a low intake of salt, refined sugar, saturated and trans fats, and carbohydrates with a low glycemic load [99,196]. This corresponds to a high intake of fruits, vegetables, legumes, fish and seafood, nuts, seeds, whole grains, vegetable oils (primarily extra virgin olive oil), and dairy foods, and a low intake of sweets, soft drinks, and red and processed meat [197,198]. Concretely, in a recent meta-analysis, which included 86 cross-sectional and 10 cohort prospective studies for a total population of more than 130,000 vegetarians and 15,000 vegans (plant-based diet, complete abstention from meat and meat products, poultry, seafood, and consumption of any other product from an animal), it was found that vegetarians and vegans had significantly lower levels of BMI, total cholesterol, LDL-cholesterol, and glucose than omnivores. Additionally, an analysis of prospective studies showed a 25% pooled significant risk reduction in ischemic heart disease incidence and/or mortality [199,200].

Conversely, the published literature generally does not support statistically significant associations between dietary cholesterol and CVD risk. The heterogeneity in the adjustment for total energy, other dietary components, and serum cholesterol concentrations shows that studies of dietary cholesterol have been conducted over a long time span during which nutritional epidemiology methods have significantly changed. Nonetheless, it has been demonstrated by Zhong et al. that there is a positive correlation between dietary cholesterol and egg consumption and the risk of CVD. According to the authors’ calculations, the risk of CVD increased significantly with every additional half egg consumed each day. However, when dietary cholesterol was taken into account, the link between egg consumption and CVD was eliminated [201]. Table 1 provides further references for all of the above and specifically for the benefits of plant-based or vegetarian diets.

Implementing various strategies that resulted in a marked decrease in inflammation diseases and consequently CVD, with an enormous increase in life expectancy as a result, was one of public health’s greatest achievements. However, over the past few decades, more and more populations have been exposed to a Western lifestyle, which is associated with a high consumption of processed foods high in calories, which has resulted in an increase in diseases that are largely preventable and linked to a chronic inflammatory state [7]. In fact, we face a pandemic of diseases linked to lifestyle because more than one third of adults worldwide are considered overweight or obese. As society becomes increasingly health-conscious, healthy dietary patterns may increase. In the upcoming years, formal dietary recommendations by major health organizations will ultimately be guided by newly emerging data highlighting new definitions of a healthy diet and which foods make up this diet. In the coming decades, the current trend towards healthier choices may extend life expectancy and improve cardiovascular outcomes [172,209].

## 10. The Effect of Western Diet on Mental Health

The bidirectional communication that maintains the microbiota–gut–brain axis is carried out through three main axes, and nervous, immune, and endocrine signaling pathways are involved [210]. This communication is carried out through the transmission of information from neurotransmitters and through the bloodstream, including metabolites, cytokines, and hormones [211]. The nervous system (NS) participates in this essential interaction, as well as the neuroimmune and neuroendocrine systems. The enteric nervous system (ENS) is a structure that is part of the autonomic nervous system (ANS), especially qualified for the regulation of gastrointestinal functions [212]. It is the most complex system of the ANS, and among its most important functions we find the regulation of the esophagus and stomach, and colorectal functions. The latter are intimately associated with digestion and nutrient absorption. In addition, it protects these cellular structures from inflammation of the organs that are part of the digestive system [213]. The ENS is called the second brain, as there are about 100 million neurons in this area, making it the largest presence of these specialized cells outside the brain [214]. Communication with the CNS is through the sensory neurons that provide information about the intestine and are connected by the vagus nerve and the motor neurons that send information to the intestine [215].

In this axis, the microbiota performs regulation of the concentration of bacterial families along the intestine as a protective action, trophic functions, metabolic functions, and nutritional functions. Due to this involvement, the microbiota is associated with the regulation of mood and cognitive functions, so it is possible to affirm its impact on the presence of mental disorders [216]. When there is an alteration in the microbiota, various physiological effects appear, such as systemic inflammation or chronic low-grade inflammation. Systemic inflammation favors neuroinflammation through different very complex mechanisms that maintain a constant interaction [217]. This inflammation is characterized by an increased presence of macrophages in peripheral cellular tissues, and an increased level of inflammatory cytokines in the blood [218]. Similarly, although low-grade systemic inflammation does not in itself cause loss or damage to the functions of the infiltrated tissues, it does facilitate vulnerability to psychopathologies directly associated with CNS functioning [219]. When this inflammation exists, microglia, which are cells that are part of the immune system, are activated, and this activation causes neuroinflammation [220].

In relation to the study of mental disorders associated with alterations in the communication of the microbiota–gut–brain axis, it has been determined that in the presence of stress disorders, patients present elevated levels of proinflammatory cytokines [221]. In this sense, mast cells, important effector cells, translate stress signals and release neurotransmitters and cytokines, and these actions have a negative impact on the patient’s anxiogenic symptomatology [222]. In addition, these patients present microinflammation of the gut mucosa and mast cell hyperplasia, which explains the maintenance of symptoms [223]. This alteration in the intestinal microbiota, called dysbiosis, is associated with the consumption of the Western diet due to its proinflammatory action [224].

Similarly, it is known that patients with mental pathologies present a higher risk of cardiometabolic diseases [225]. The comorbidity of mental disorders with metabolic syndrome (MS) is already a reality, with an incidence of 60% in these patients. Although it was initially thought that MS could appear because of the pharmacology associated with psychopathology, we now know that patients with depressive disorder, anxiety disorder, psychotic disorder, and bipolar disorder who do not take drugs to treat these also have a higher risk of suffering from MS [226,227].

It is precisely for this reason that it is essential to understand that there are pathophysiological elements that function as connecting structures between these pathologies. In psychiatric patients, MS can appear due to the acquisition of unhealthy nutritional habits, as well as the consumption of alcohol, tobacco, irregular sleep behaviors, and sedentary lifestyles [228]. At the molecular level, psychiatric patients seem to present definite characteristics that may make them more vulnerable to MS, for example, a modification in the sensitivity to glucocorticoids, which implies a dysregulation of the hypothalamic–pituitary–adrenal (HHA) axis and is caused by high levels of stress in the organism [229]. This high adrenocortical stimulation favors dyslipidemia, an alteration in the circulation of lipids in the blood [230].

Moreover, the presence of certain pleiotropic genes that seem to be involved in the connection between mental pathologies and cardiac and metabolic conditions has been studied with great interest in recent years [231,232]. These studies have shown that psychiatric patients have an increased risk of MS. This can be explained by the fact that serotonin 2C receptor genes and obesity-associated genes such as leptin are involved in the pathogenesis of both diseases [233,234]. The role of the microbiota has also been studied in terms of its involvement in modulating the production of signals associated with neuronal plasticity [235].

In the case of metabolic and endocrine disturbances and depression, we can see that recent studies have shown that the effects of stress act on the response of the hypothalamus by releasing corticotropin and vasopressin, which causes corticotropin-bearing neurons to project to noradrenergic centers and stimulate the spinal cord [236]. This promotes the action of sympathetic branch neurons through the stimulation of α1-adrenergic receptors and, in turn, this stimulates corticotropin release in the hypothalamus, creating a positive bidirectional feedback loop [237]. In recent years, research on intestinal microorganisms that can cause neurological problems and psychopathologies has been increasing, thanks to the knowledge we now have about the functioning of the bidirectional microbiota–intestine–brain axis [238]. Previous studies have been able to show that in patients with depression, there are two types of bacteria whose levels are low or even non-existent, Dialister and Corproccus. In this sense, it is known that certain nutrients have an antidepressant action; among the most important, we find the long-chain omega-3 fatty acids (EPA and DHA), magnesium, potassium, iron, and vitamins B6, B12, A, and C [239,240].

Currently, the incorporation of prebiotic and probiotic products in the diet is presented as a beneficial strategy for people at risk of suffering a mental disorder or people who have been diagnosed with a psychopathology [241,242,243]. In fact, these foods are now considered to be psychobiotics. Prebiotics are products that nourish the growth of some beneficial bacterial species such as lactobacilli and bifidobacteria. Starch, which promotes insulin sensitivity and mobilizes fats in the process of obtaining energy, is also present in this group [244,245].

Similarly, inulin and galacto-oligosaccharides (GOS), in addition to improving the cholesterol profile presented in animal [246] studies, increase the attentional level as well as improve cognitive processing. In addition, they decrease cortisol levels, which has a positive impact on the presence of mood disorders. On the other hand, the Lactobacillus Casei Shirota family of bacteria can inhibit the proliferation of harmful bacteria, in addition to increasing the immune response [247]. In its interaction in the gut–brain axis, it reduces anxiety levels, decreases chronic fatigue, and improves mood [248,249].

With regard to probiotics, the formula Lactobacillus helveticus and Bifidobacterium longum results in a significant reduction in psychological anxiety, improved emotional well-being, and a reduction in the vagus activation of the sympathetic branch [250,251]. Another formulation that has beneficial effects on mental health are species-specific formulations of lactobacilli and bifidobacteria, called friendly bacteria, a group of saccharolytic bacteria that produce short-chain fatty acids such as lactate and acetate [252,253]. This composition has been shown to reduce the symptoms associated with depression and negative and aggressive emotions [254].

After reviewing the Western diet, it is possible to affirm that its effects on general health, and more specifically on mental health, are not beneficial. The food composition of this diet, rich in processed foods, red meat, and saturated fats among others, as well as the lack of fiber and vitamins, makes the body vulnerable to the presence of mental pathologies. Among the most important are mood disorders in the face of chronic low levels of inflammation, which appear as a defense and repair mechanism in the face of intestinal dysfunction. Systemic inflammation increases the circulation of inflammatory cytokines and the passage of macrophages in cellular tissues, which facilitates the activation of other dendritic cellular inflammation processes that can be found in neurodegenerative diseases, although it remains to be confirmed whether this inflammation is a cause or effect of the pathology.

## 11. The Effect of Western Diet on Metabolism

Diabetes, including hyperinsulinemia and insulin resistance, obesity, hyperlipidemia, and cardiovascular disease have been extensively linked to the Western diet [10,77].Recent literature has highlighted how the Western diet may contribute to systemic inflammation enhancement, due to the large amount of fats present in the Western diet, as well as it effects triggering oxidative stress [255].

Considering both the quantity and quality of macronutrients, it is intriguing to note how the Western diet may affect general health in this passage. In terms of quantity, the Western diet has been associated with increased intakes of ultra-processed foods, such as sweets, soft beverages, processed meats, refined potato and maize products, and high-fat dairy products, as well as increased intakes of animal fats. In addition, it has been noted that the Western diet was associated with a lower ingestion of unprocessed fruits, vegetables, seeds, and whole cereals, as well as fish [12,197,256,257]. With regard to quality aspects, the Western diet has been related to elevated saturated and omega-6 polyunsaturated fatty acid consumption as well as to a decreased omega-3 polyunsaturated fatty acid intake, which may compromise cardiovascular condition [258,259]. However, Nicholls et al. note that not all evidence points in the same direction; in patients with a high cardiovascular risk, the addition of omega-3 to standard background regimens did not result in a significant difference in the composite outcome of severe adverse cardiovascular events when compared to maize oil. These results do not support the use of this omega-3 fatty acid formulation to prevent severe cardiovascular adverse events in patients at high risk [260].

Moreover, regarding a lower fruit and vegetable intake, the latest literature proposed that this could be related to a decreased fiber and antioxidant exposition, which is present in this type of food, avoiding their benefits for the general health status [261]. Similarly, the recent literature proposed that reduced fruit and vegetable consumption has been inversely associated with metabolic syndrome, supporting the protective role of these foods in overall health management [262]. In a study by Li et al. combining a fruit and vegetable diet with physical activity(PA), metabolic syndrome (MS) was prevalent in 28.7% of participants, with prevalence rates of 24.7% in men and 32.9% in women. Compared to participants with inadequate PA and inadequate FV intake, those with adequate PA and adequate FV intake had the lowest risk of MS [263].

In order to improve the comprehension of the Western diet in health, it is necessary to understand the key role of inflammation, which may contribute enhancing the pathogenesis of metabolic syndrome [264]. MS, also known as insulin resistance syndrome or syndrome X, is a complex pathology due to the phenotypic profile that presents with wide variations and is associated with the acquisition of unhealthy nutritional habits, sedentary lifestyles, and obesity [265].Thus, pro-inflammatory substances increase oxidative stress, triggering different pathologies associated with metabolic syndrome, such as type 2 diabetes, dyslipidemia, obesity, atherosclerosis, cardiomyopathy, hypertension, and heart failure [266,267,268]. Hence, previous authors proposed that these findings may be explained by the fact that elevated saturated fatty acid consumption may be related to postprandial inflammation [269]. The postprandial state is defined as the moment that occurs after food ingestion, lasting between 6 and 12 h, in which an increase in carbohydrates and fatty acids occurs [270]. Furthermore, the postprandial state may be considered as a source of pro-inflammatory substances, since previous authors proposed that saturated fatty acids may activate the immune response [271,272]. These studies proposed that saturated fatty acids may present analogous molecular effects to lipopolysaccharide, a molecule which is responsible for Toll-like receptor 4 stimulation, which consequently promotes pro-inflammatory cytokine liberation and disturbs cellular metabolism. Additionally, previous authors pointed out that polyunsaturated fatty acids may also have a great impact on metabolic diseases, since omega-3 fatty acids were related to postprandial inflammation suppression, whereas omega-6 fatty acids were linked to inflammatory processes [273,274,275,276,277,278]. These outcomes were also supported by the recent and previous literature, in which positive associations were found between type 2 diabetes, atherosclerosis, non-alcoholic fatty liver disease, and postprandial state [279,280,281], suggesting the importance of postprandial inflammation and its relationship between different diseases that compound metabolic syndrome. In relation to this, a review of the use of citrus fruits notes that hesperidin, hesperetin, naringenin, naringin, and narirutin have anti-inflammatory effects in model systems, and human trials with hesperidin report a decrease in inflammatory markers. Orange juice reduced inflammation caused by a high-fat, high-carbohydrate meal in humans [282].

Regarding fiber, the previous literature suggested that patients who followed the Western diet were more likely to suffer obesogenic surroundings, increasing visceral obesity growth and insulin resistance [283]. According to these findings, diets that involved a raised fiber intake were related to a decreased cardiovascular disease, metabolic syndrome, and gastrointestinal disease prevalence [284]. The beneficious role of fiber may be explained through its capability to modulate inflammatory and proliferation processes as a result of its effect in the gastrointestinal tract. Thus, when fibers are introduced into an organism, they are not digested in the gastrointestinal tube, but rather fermented by gut microbiota, generating short-chain fatty acids, which play an important role as anti-inflammatory molecules [285,286]. For instance, after conducting a randomized controlled trial with a 3-week intensive diet-exercise intervention, Tremblay et al. concluded that adequate dietary fiber intake should be a primary factor in diet-based weight loss programs [287].

These findings suggest that fiber may be an effective weapon against metabolic disorders by virtue of its ability to lower oxidative stress. Antioxidant chemicals are molecules that have the capacity to reduce oxidative stress and, as a result, may have a beneficial influence on health. Polyphenols, which have been shown to have anti-oxidative and anti-inflammatory properties, have been suggested as a potential tool in the prevention and control of diabetes [288]. Additionally, the previous literature proposed that the use of flavonoids, present in citrus products, may have antioxidant properties since the citrus flavonoid narangin may reduce proinflammatory substances such as TNF-a and cyclooxygenase-2, as well as inducible NO synthase activity [289]. Considering these findings, citrus flavonoids seem to be beneficious for metabolic syndrome and obesity treatment [290], as they perform an important role as an antioxidant, reducing inflammatory processes. With regard to fatty acids and their effect on oxidative stress, the latest literature proposed that a higher ratio between omega-6 and omega-3fatty acids was strongly related to greater mortality from all causes of death, cancer, and cardiovascular disease [291]. These results may be explained by the fact that omega-6 fatty acids have strong pro-inflammatory activity while omega-3 polyunsaturated fatty acids present antioxidant effects. Furthermore, it has been largely proposed that the Mediterranean diet, in contrast to the Western diet, is composed of several antioxidant substances, such as oleic acid, a mono-unsaturated fatty acid that can be found in olive oil, as well as alpha- linolenic acid, an omega-3-polyunsaturated fatty acid present in nuts and fish such as salmon, tuna, or sardines [292,293,294]. Finally, it has been reported that this kind of diet also involves a raised amount of polyphenols such as flavonoids, present in fruits and vegetables, as well as a substantial quantity of fiber, with all these being antioxidant components able to enhance health status by reducing oxidative damage [295]. Thus, considering these outcomes, it could be proposed that the Western diet may have a negative impact on metabolic syndrome.

## 12. The Effect of Western Diet on Cancer

The impact of the Western diet on noncommunicable diseases, including its negative effects on cancer, has been extensively studied. However, there has been conflicting evidence from research looking at the link between specific foods and diseases, and even fewer studies have been carried out on diet as a whole, with conflicting results [296]. Nevertheless, the Western diet could be considered a dietary choice that may compromise health status by promoting inflammation and increasing the risk of developing these types of pathologies, as well as increasing their mortality and morbidity [12,297,298].

With regard to cancer, several studies pointed out that it may be modulated by different molecular processes, including oxidative stress and inflammation [299]. Similarly, previous authors related the raised production of reactive oxygen species (ROS) to numerous different types of cancer, due to the raised oxidative stress suffered by cells [299]. For instance, the utilization of endogenously produced reactive oxygen species (ROS) through the activation of signaling pathways including PI3K/AKT and MAPK can also contribute to the development of ovarian cancer [300]. This fact may be explained through ROS’ capability of promoting the initiation of oncogenes as well as by their ability to reduce tumor suppressor activity. Moreover, ROS play a crucial role in inflammatory response, since they have been associated with a decrease in p53 levels, with p53 being one of the most important tumor suppressors that can slow cancer progression. Additionally, increased ROS levels were related to a significant increase in cytokine levels, which may compromise the inflammatory response [301]. Subsequently, the previous literature has related the elevation of cytokine levels to an increased tumor proliferation as well as to cancer progression [302,303], compromising cancer management and survival.

The transition from whole foods to processed ones in the Western diet triggers a reduction in the micronutrient quality of this kind of diet [304]. However, fruits and vegetables with significant antioxidant content may help prevent malignancies of the mouth, pharynx, larynx, esophagus, stomach, and lungs, according to the available research on malignancies of the lungs, esophagus, stomach, and larynx [305].

Thus, the absence of these foods prevents individuals from obtaining nutrients that can be beneficial, whereas they consume saturated fats and refined sugar, which may negatively affect their overall health due to their pro-inflammatory effects. Regarding lipid consumption, the recent literature proposed that individuals with lower levels of omega-3 polyunsaturated fatty acids presented a 30% greater risk of cancer mortality [291,306], suggesting the protective effect that omega-3 polyunsaturated fatty acids may have in cancer development due to their capability modulating oxidative stress and inflammation response. With regard to specific types of cancer, a recent metanalysis noted that lower risk of developing colorectal cancer has been associated with raised docosahexanoic acid (DHA) and eicosapentaenoic acid (EPA) intake, both omega-3 polyunsaturated fatty acids, as well as with decreased linoleic acid intake, an omega-6 polyunsaturated fatty acid [307]. Furthermore, colorectal cancer also has been associated with an elevated trans fatty acid intake [308], a special kind of fatty acid that suffers a partial hydrogenation in the industrial process in order to deodorize and heat vegetable oils at great temperatures [309]. Thus, trans fatty acids may have a negative effect on health due to their potentially pro-inflammatory activity, enhancing cytokine release from monocytes and macrophages, such as tumor necrosis factor-α (TNF-α), interleukin-6 (IL-6), and monocyte chemoattractant protein (MCP) [310,311]. Not only has it been found that industrial trans fatty acids are linked to an increased risk of colon cancer, industrial TFAs were also shown to have a stronger correlation with colorectal cancer [312].

Concerning the current refined sugar intake in the Western diet, it is important to consider carbohydrates’ effects on tumoral events, since the latest studies proposed the high glucose levels could be related to an improvement in vascular endothelium growth, as well as to the reduction in anti-angiogenic factors [313]. This could be explained by the fact that cancer cells might enhance their growth and proliferation when they are in a nutrient-rich environment, as was largely described several years ago by Otto Warburg, who considered that cancer cells may obtain energy through aerobic glycolysis [314]. According to these findings, it may be considered that an increased refined sugar intake may be associated with a worse efficacy in tumoral events, since several studies proposed that higher simple carbohydrate intake may be related to a worse cancer prognosis [315,316,317,318,319]. Therefore, these outcomes support the idea that the Western diet, which involves a raised simple carbohydrate intake, may have a negative impact on cancer disease. For instance, breast cancer risks were shown to rise by more than 10% for every 10% increase in the amount of ultra-processed foods in the diet in a large prospective investigation [320].

Regarding fruit and vegetable consumption, several authors suggested their intake may have a beneficious outcome in cancer, due to their higher fiber and complex carbohydrate content. More specifically, reduced fruit and vegetable ingestion has been inversely correlated with a raised risk of developing different types of cancer, including lung cancer [321]. This may be explained by the ability of complex carbohydrates to modify insulin-like growth factor binding protein 3 (IGFBP-3) activity. This protein has been shown to be responsible for insulin/IGF-1 axis disturbance, blocking cell proliferation and tumoral growth, which may control cancer development. Additionally, both complex carbohydrates and fiber could modify the gut microbiota, and as mentioned above, they might modulate short-chain fatty acid production from the gut microbiota, decreasing inflammation and promoting tumoral control. Lastly, the potential effect of complex carbohydrates and fiber on excretion processes has been highlighted, since they might be liable for raising carcinogen fecal excretion, enhancing their elimination and reducing their time inside the organism, and, consequently, their associated negative effects [322]. Finally, considering the antioxidants present in fruit and vegetables, several studies pointed out their benefits as cancer-modulating molecules, due to their ability to reduce cellular stress and, consequently, modulate tumorigenesis through modifications in gene expression [323,324,325]. Interestingly, the highest values for antioxidant substances were found in fruits, such as berries, as well as in nuts, including walnuts, sunflower seeds, and pecans [326,327]. Regarding nuts, it has been widely described that they may constitute an advantageous tool in order to modulate cancer development. Thus, their polyphenols, including ellagic acid, anacardic acid, resveratrol, and inositol, among others, may be related to apoptosis modulation, cell growth, migration and invasion inhibition, and angiogenesis and metastasis control [328,329,330,331,332].

Therefore, taking into account that the Western diet avoids this type of nutrients, it would be considered that individuals who follow this type of diet are less likely to benefit from these phytochemicals and their intake.

## 13. Sanitary Costs of Western Diet

There are several pathologies associated with the different risk factors associated with the consumption of the Western diet, such as obesity and type 2 diabetes [333,334,335]. In this regard, obesity has tripled over the last 5 years. According to the Organization for Economic Cooperation and Development (OECD), in a report made in 2019 [336], it is predicted that within 30 years, obesity or overweight is going to be triggering the onset of 70% of cases of patients diagnosed with diabetes, around 20% of heart disease, and 9% of cancer cases. This means that the treatment of obesity and associated diseases is not only a public health problem but also a very high economic cost for societies [336,337].

According to data collected in another study conducted by the World Obesity Federation (WOF) in 2022 [338], it is possible to predict that in 2030, one in five women and one in seven men will be obese. This projection is generalized for all countries in the world. In addition, approximately 14% of the child population will be obese. This means that around one billion people will suffer from obesity worldwide [338,339]. From the numbers collected in relation to the cost involved, we can see that in Australia, USD 24 billion is spent annually on the treatment of these pathologies. In Brazil, the annual expenditure is USD 39 billion. In India, this expenditure amounts to USD 23 billion a year. Although these figures are already alarming, a very significant increase is expected within a few decades [339].

Type 2 diabetes is a disease associated with a high rate of comorbidity and mortality. Both incidence and prevalence have increased in recent years, with a greater presence in developed countries and associated with sedentary lifestyles, obesity, and increased life expectancy. The organic degeneration it entails makes it serious because of its morbidity and mortality. It is, therefore, a major public health problem worldwide [340].

Sources consulted indicate that the prevalence worldwide is approximately 8.5%, and a growth in this range is expected by 2035, exceeding 590 million people with obesity [341,342] as a high-impact factor. In the US, about 15.5 million people have type 2 diabetes and at least 13 million people have impaired glucose tolerance. In Europe, chronic and acute complications appear in more than 10 million people. In Asian countries, there has been a rapid increase in the incidence, mainly due to the change in nutritional habits during the last few decades [342].

In relation to comorbidity, it is estimated that the chronic complications that occur in this pathology because of its vascular degenerative course are the most frequent [343]. Among the most important are heart failure, peripheral and cerebral vascular disease, retinopathy, and neuropathy [344,345]. Another of the highest costs is hospitalization because of the disease, hyperglycemia, hypoglycemia, infections, exacerbations, and/or ketoacidosis, among others. Hypoglycemia is frequently a reason for consultation that is also associated with a reduction in or loss of work [346]. Its incidence is around 75 cases per 1000 patients per year after 5 years of diagnosis. Associated diseases such as intestinal complications and/or nasal and pharyngeal infections are also a reason for consultation and hospitalization, accounting in some countries for up to 20% of the costs associated with the disease [347].

The healthcare costs incurred in 2013 worldwide indicate that approximately USD 550 million was spent on the treatment and follow-up of these patients [348]. This includes direct healthcare costs, non-direct healthcare costs related to aspects such as the number of healthcare personnel needed, transportation costs, and care services, among others, and costs due to the loss of productivity, which refer to work incapacity, absenteeism, and low productivity, among others [349]. Finally, intangible costs are included, which refer to the reduction in or even loss of well-being, both of patients and their immediate environment, associated with suffering and pain [350,351].

Metabolic syndrome, also known as insulin resistance syndrome or syndrome X, is a complex pathology due to the phenotypic profile that presents with wide variations and is associated with the acquisition of unhealthy nutritional habits, sedentary lifestyles, and obesity [352]. It is a pathology in which different metabolic and inflammatory alterations are present that favor the appearance of cardiovascular pathologies [353]. Statistics reveal that, when stratified by diabetes, the average yearly total expenses of participants with and without metabolic syndrome varied by an overall magnitude of 1.6 (USD 5732 vs. USD 3581) and an overall magnitude of 1.3 (USD 7896 vs. USD 6038; USD 4476 vs. USD 3422), respectively. Per extra risk factor, overall expenses rose by an average of 24% (*p* = 0.001). Costs for patients with diabetes who also had weight risk, dyslipidemia, and hypertension were nearly twice as expensive (USD 8067 vs. USD 4638) as those for patients with prediabetes who also had these risk factors [354].

The prevalence in adults in the US stands at almost 23% for both men and women. In Europe, this prevalence is somewhat lower, being 10% in women and 15% in men. In recent years, the incidence has increased significantly and in relation to obesity [355]. With regard to the health costs associated with metabolic syndrome, there are also those derived from treatments for obesity, type 2 diabetes, hypertension, and hypercholesterolemia. All this means that this cluster of pathologies represents a very high annual cost. For this reason, in 2004, the WHO published a document called Global Strategy on Diet and Physical Activity for Health, with the aim of implementing strategies and actions to reduce the impact of these diseases on society. In 2011, the United Nations General Assembly scheduled a meeting in which it adopted a resolution called “Political declaration of the high-level meeting of the General Assembly on the prevention and control of non-communicable disease”, with the same objective of prevention [356].

Cardiovascular disease is the leading cause of death in the world. It affects all countries equally and is associated with the presence of risk factors such as unhealthy eating habits, obesity, and hypertension, among others [357]. These factors are present in a large part of society due to the general lack of physical activity, as well as the habit of hypercaloric diets with low consumption of vitamins and fiber, so its consequences worldwide are very serious. Among the most important are coronary heart disease, cerebrovascular disease, rheumatic heart disease, deep-vein thrombosis, and pulmonary embolism [358]. Approximately 18 million people worldwide died from this cause in 2015, accounting for more than 30% of all recorded deaths, being above deaths from cancer, HIV, or respiratory system infections. The recorded data indicate that the costs of these pathologies are the highest in the world. In the United States, more than USD 450 billion is spent annually on treatment and associated indirect costs [358]. In Europe, more than 20% of people admitted to hospitals are admitted for these diseases, and the associated cost exceeds EUR 170 billion per year. In countries such as France, Germany, and the United Kingdom, the direct cost of these pathologies amounted to more than EUR 100 billion in 2014, a figure comparable to the GDP of some small or medium-sized countries [359]. In addition, the indirect costs involved must be added to this, the psychological and other costs associated with the triggers and diagnosed pathologies [360,361].

Cancer is another pathology associated with the Western diet. Related to metabolic syndrome and obesity, it is known that the presence of these characteristics causes changes in an organism that facilitate the appearance of certain cancers [362,363]. As a result of these changes, chronic inflammation may appear, increasing insulin levels. Studies conducted in 2014 indicated that the increase in cancers in the US related to obesity and overweight was 7%, recording in contrast that cancers associated with other factors decreased by 13% [364,365]. The figures indicate that nearly 700,000 people a year are diagnosed with obesity-related cancer [366].

In addition, the occurrence of diseases associated with the inflammation and infection of the intestinal system should be considered [367]. In this regard, inflammatory bowel disease has an unknown prevalence, although it appears more frequently in developed societies [368]. Among the most important of this group of pathologies, we find Crohn’s disease and ulcerative colitis. With regard to Western countries, there is a higher incidence of diagnosis of Crohn’s disease in the US, with figures of 20.2 per 100,000 inhabitants [369]. In Europe, however, there is a higher incidence of ulcerative colitis, standing at 24.3 per 100,000 people [370]. It is interesting to see how in Asia, Africa, and the Middle East, a few years ago, very low prevalence figures were recorded, although an increase in the diagnosis of inflammatory bowel disease seems to be detected, mainly associated with the social and economic development of some specific countries [371].

These diseases currently have no known cure, so treatment is a matter of indefinite follow-up, with the intention of keeping the patient in remission [372,373]. This means that once the diagnosis is made, we will have these people in follow-up consultations, check-ups, and pharmacology throughout life, which is associated with high healthcare costs during this process [374,375,376,377,378,379,380]. In addition, in these diseases, we must consider that there will be moments of remission, but also episodes in which the symptomatology worsens significantly during outbreaks [381,382,383].

The annual health costs in all developed countries are currently very high if we consider the pathologies associated with the consumption of the Western diet. This diet, which includes an excess of calories and the intake of foods with pro-inflammatory action such as polyunsaturated fats and sugar, favors the presence of various diseases by itself or as a cause of other associated pathologies. These facts allow us to understand the social and economic repercussions that are involved with the habit of this style of nutrition. This should be enough for institutions to introduce actions and measures aimed at a correct understanding of the characteristics of the Western diet and its risks for the population, as well as actions aimed at preventing the appearance of organic pathologies. Special mention should also be made of the current situation of overcoming the COVID-19 pandemic, where these factors only increase the risk for this type of pathology and other related ones [384,385,386].

## 14. Practical Applications

Improving the Western diet and lifestyle can have a significant impact on health outcomes, reducing the risk of chronic diseases such as heart disease, diabetes, and certain types of cancer. We can propose some practical applications that can help improve the Western diet and lifestyle:

- Increase intake of fruits and vegetables: A diet rich in fruits and vegetables provides a range of vitamins, minerals, and antioxidants that can promote health and reduce the risk of chronic diseases. Encouraging people to eat a variety of colorful fruits and vegetables and including them in meals and snacks can help increase their intake.
- Reduce intake of processed and fast food: Processed and fast foods tend to be high in calories, unhealthy fats, sugar, and sodium and low in nutrients. Encouraging people to cook meals at home, emphasizing the importance of reading food labels, and promoting healthier fast food options can help reduce intake.
- Encourage physical activity: Regular physical activity is essential for maintaining a healthy weight and reducing the risk of chronic diseases. Encouraging people to find enjoyable ways to be active, such as walking, biking, or dancing, and emphasizing the importance of incorporating physical activity into daily routines can help increase physical activity levels.
- Limit sedentary behavior: Sedentary behavior, such as sitting for long periods of time, can increase the risk of chronic diseases. Encouraging people to take breaks from sitting, such as standing or walking, and promoting workplace wellness programs can help reduce sedentary behavior.
- Promote mindful eating: Mindful eating involves paying attention to the present moment and being aware of food choices, hunger, and fullness cues. Encouraging people to eat slowly, savor their food, and listen to their body’s cues can help promote mindful eating.
- Emphasize the importance of sleep: Getting enough sleep is important for overall health and well-being. Encouraging people to prioritize sleep and establish healthy sleep habits, such as going to bed and waking up at consistent times, can help improve sleep quality.
- By incorporating and enhancing psychometric evaluation questionnaires specifically designed for assessing one’s lifestyle choices, health professionals will be empowered to provide more personalized and targeted interventions.

Overall, these practical applications can help improve the Western diet and lifestyle, promoting better health outcomes and reducing the risk of chronic diseases.

## 15. Conclusions

**Figure 5:**
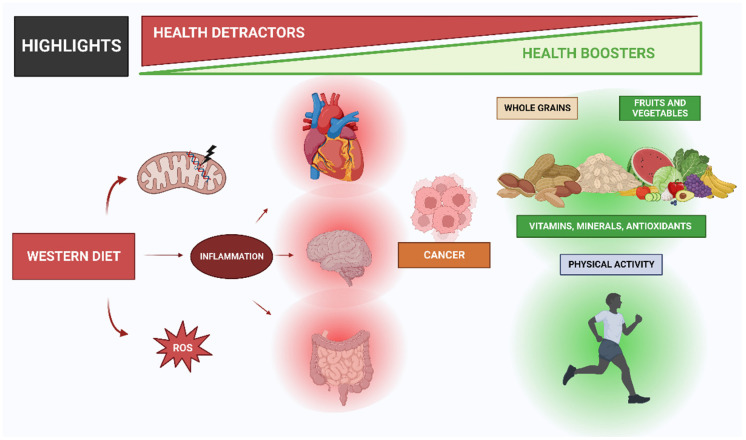
Key highlights from this analysis of the Western diet that emphasize its negative effects on health and suggestions for improving these effects on your body.

This review aimed to describe the effects of the Western diet on the metabolism, inflammation, and antioxidant status, as well as on the gut microbiota and mitochondrial fitness, cardiovascular and mental health, and cancer, in addition to the Western diet’s hygienic costs. As presented in Figure 5, the most important highlights of this review are, firstly, that fruits, vegetables, fiber, and complex carbohydrates can combat not only metabolic disorders that an individual may be afflicted with, but also cancer and its development in relation to the consumption of typical Western diet foods. Secondly, in this regard, consuming a healthier diet rich in micronutrients and antioxidants such as magnesium, potassium, iron, vitamins B6, B12, A, and C, carotenoids, and flavonoids as well as engaging in physical activity reduce inflammatory processes, which has consequences such as improving mental health, increasing beneficial bacteria in the gut, enhancing mitochondrial function, and boosting our immune system.

## Figures and Tables

**Table 1:** Contributions from references pointing to cardiovascular improvements in individuals following a diet without the Western diet patterns.

	Study Design	Ethnic Majority Population, *n*	Study Participants	Type of Diet	Results
Shah et al. [202]	Randomized Controlled Trial	White, *n* = 100	Male and female, 63 years, 30.5 kg/m^2^	Vegan	Lower *C*-reactive protein
Djekic et al. [203]	Randomized Controlled Trial	White, *n* = 31	Male, 67 years, 28 kg/m^2^	Vegetarian	Decreased total cholesterol, LDL-C, and body weight
Djekic et al. [204]	Randomized Controlled Trial	White, *n* = 31	Male, 67 years, 28 kg/m^2^	Lacto- ovo vegetarian	Increased plasma lipid profiles (TGs, PCs, O-PCs, and SMs)
Mishra et al. [205]	Randomized Controlled Trial	White, *n* = 291	Male and female, 44 years, 34.7 kg/m^2^	Plant-based diet	Decreased total cholesterol and LDL-C
Turner-McGrievy et al. [206]	Randomized Controlled Trial	Black, *n* = 159	Male and female, 48 years, 25 kg/m^2^	Plant-based diet	Decreased total cholesterol, LDL-C, and body weight
Wright et al. [207]	Randomized Controlled Trial	White, *n* = 65	Male and female, 46 years, 34.5 kg/m^2^	Plant-based diet	Decreased cholesterol and BMI
Jenkins et al. [208]	Randomized Controlled Trial	White, *n* = 39	Male and female, 55 years, 30.5 kg/m^2^	Low-carbohydratesdiet	Total cholesterol and LDL-C decreased

## Data Availability

Not applicable.
